# Identification of Xanthomicrol as a Major Metabolite of 5-Demethyltangeretin in Mouse Gastrointestinal Tract and Its Inhibitory Effects on Colon Cancer Cells

**DOI:** 10.3389/fnut.2020.00103

**Published:** 2020-07-29

**Authors:** Xian Wu, Zhengze Li, Yue Sun, Fang Li, Zili Gao, Jinkai Zheng, Hang Xiao

**Affiliations:** ^1^Department of Food Science, University of Massachusetts, Amherst, MA, United States; ^2^Department of Kinesiology and Health, Miami University, Oxford, OH, United States; ^3^Anhui Engineering Laboratory for Agro-products Processing, School of Tea & Food Science, Anhui Agricultural University, Hefei, China; ^4^Institute of Food Science and Technology, Chinese Academy of Agricultural Sciences, Beijing, China

**Keywords:** polymethoxyflavone, 5-demethyltangeretin, xanthomicrol, biotransformation, colon cancer

## Abstract

5-Demethyltangeretin (5DT) is a unique polymethoxyflavone mainly found in the peel of citrus, and has shown potent suppressive effects on multiple human cancer cells. Biotransformation plays a critical role in the biological activities of dietary bioactive components because their metabolites may exert significant bioactivities. In the present study, the metabolic fate of 5DT in mouse gastrointestinal (GI) tract after long-term oral intake and the anti-cancer effects of its major metabolite were determined. It was found that 5DT underwent extensive biotransformation after oral ingestion in mice. A major demethylated metabolite was produced via phase I metabolism, while conjugates (glucuronide and sulfate) were generated via phase II metabolism. Specifically, 4'-position on the B ring of 5DT was the major site for demethylation reaction, which led to the production of xanthomicrol (XAN) as a major metabolite. More importantly, the level of XAN in the colon was significantly higher than that of 5DT in 5DT-fed mice. Thus, we further determined the suppressive effects of XAN on human colon cancer HCT116 cells. We found that XAN effectively inhibited the proliferation of HCT116 cells by arresting cell cycle and inducing cellular apoptosis, which was further evidenced by upregulated p53 and p21 and downregulated cyclin D and CDK4/6 level. In conclusion, this study identified XAN as a major metabolite of 5DT in mouse GI tract, and demonstrated its suppressive effects on HCT116 colon cancer cells.

**Graphical Abstract:**
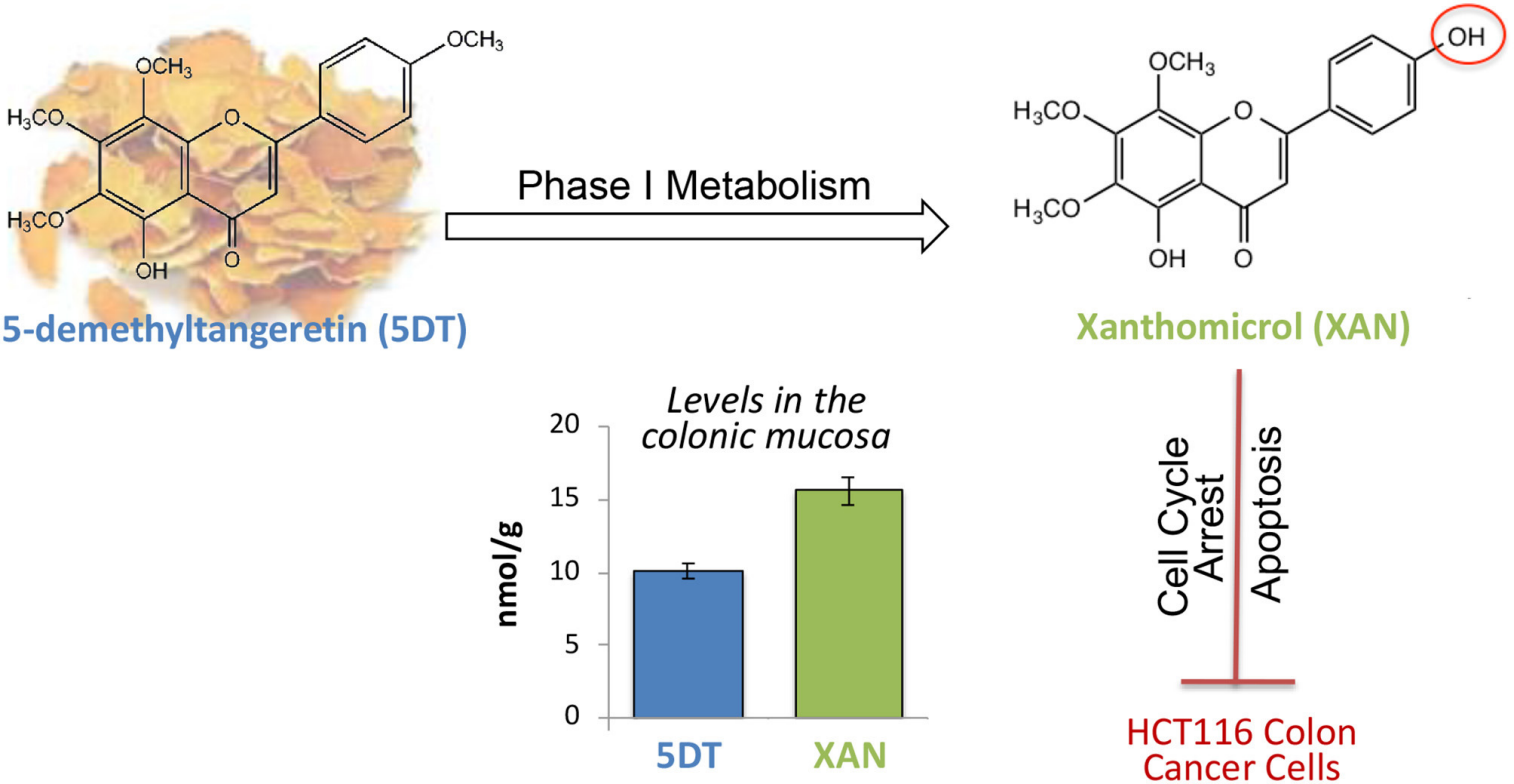
Xanthomicrol (XAN) was the major metabolite in the colon of 5-demethyltangeretin (5DT)-fed mice, and the colonic level of XAN was significantly higher than that of 5DT. Additionally, XAN potently suppressed the proliferation of HCT116 cells by arresting cell cycle and inducing cellular apoptosis.

## Introduction

Polymethoxyflavones (PMFs) are unique, natural flavonoids present in citrus fruits. Previous researches have demonstrated a broad spectrum of beneficial effects of PMFs, including antioxidant, anti-inflammation, anti-obesity, and anti-cancer effects (1). Tangeretin and nobiletin are the most common PMFs found in citrus peels (1, 2). In recent decades, a unique subclass of PMFs, hydroxylated PMFs has been characterized. Hydroxylated PMFs are mainly formed during long-term storage of citrus fruits, most of which are 5-demethylated PMFs, such as 5-demethyltangeretin (5DT) and 5-demethylnobiletin (5DN) (1, 3). Accumulating studies suggested that hydroxylated PMFs may exert more potent bioactivities than their PMF counterparts (4–10). Previously, we reported that demethylation at 5-position on the A ring significantly enhanced the growth inhibitory effects of PMFs on human colon cancer cells compared to their counterparts (7). In human non-small cell lung cancer (NSCLC) cells, 5DT was more efficacious than its counterpart tangeretin in inhibiting cancer cell proliferation and inducing apoptosis and cell cycle arrest (9).

Biotransformation may greatly affect the biological properties of dietary compounds. The metabolites generated by the biotransformation may exhibit different biological activities compared to the ingested, parent compounds (11). Some mono- and di- hydroxylated PMFs are formed during the biotransformation of PMFs (1, 12, 13). For example, 4′- and 3′- positions on the B-ring of nobiletin and 5DN were two major sites for demethylation metabolism (6, 14–16). Further, these mono- and di- hydroxylated metabolites may exert greater beneficial bioactivities compared to nobiletin or 5DN. In particular, 4′-demethylnobiletin showed more potent anti-inflammatory effects on chemical induced skin inflammation in mice than nobiletin did (5). Also, 5,3′-didemethylnobiletin, the 3′- hydroxylated metabolite of 5DN, resulted in a 49-fold stronger anti-proliferative effect on H1299 NSCLC cells than 5DN did (8). Altogether, biotransformation of PMFs, especially the formation of hydroxylated metabolites may contribute significantly to the health benefits of dietary PMFs.

However, to our knowledge, the metabolism and biotransformation of 5DT has not been systemically studied. In our recent study, we found that xanthomicrol (XAN) was an abundant metabolite of 5DT in the colonic mucosa of 5DT-fed mice (17). After 4 weeks oral intake of 5DT, the concentration of XAN was much more abundant than that of 5DT in the colonic mucosa (17). Moreover, XAN showed potent anti-inflammatory properties in lipopolysaccharide-treated RAW 264.7 macrophages, at physiologically achievable concentrations (17). However, as a major metabolite of 5DT found in the colonic mucosa, XAN's anti-cancer efficacy on colon cancer cells has never been reported. Herein, we characterized the metabolic fate of 5DT in mouse GI tract (stomach, small intestine, cecum, and colon) after long-term oral intake, and determined the anti-cancer effects of its major colonic metabolite XAN in human colon cancer HCT116 cells.

## Materials and Methods

### Animals, Diets, and Experimental Procedure

**Figure 1:**
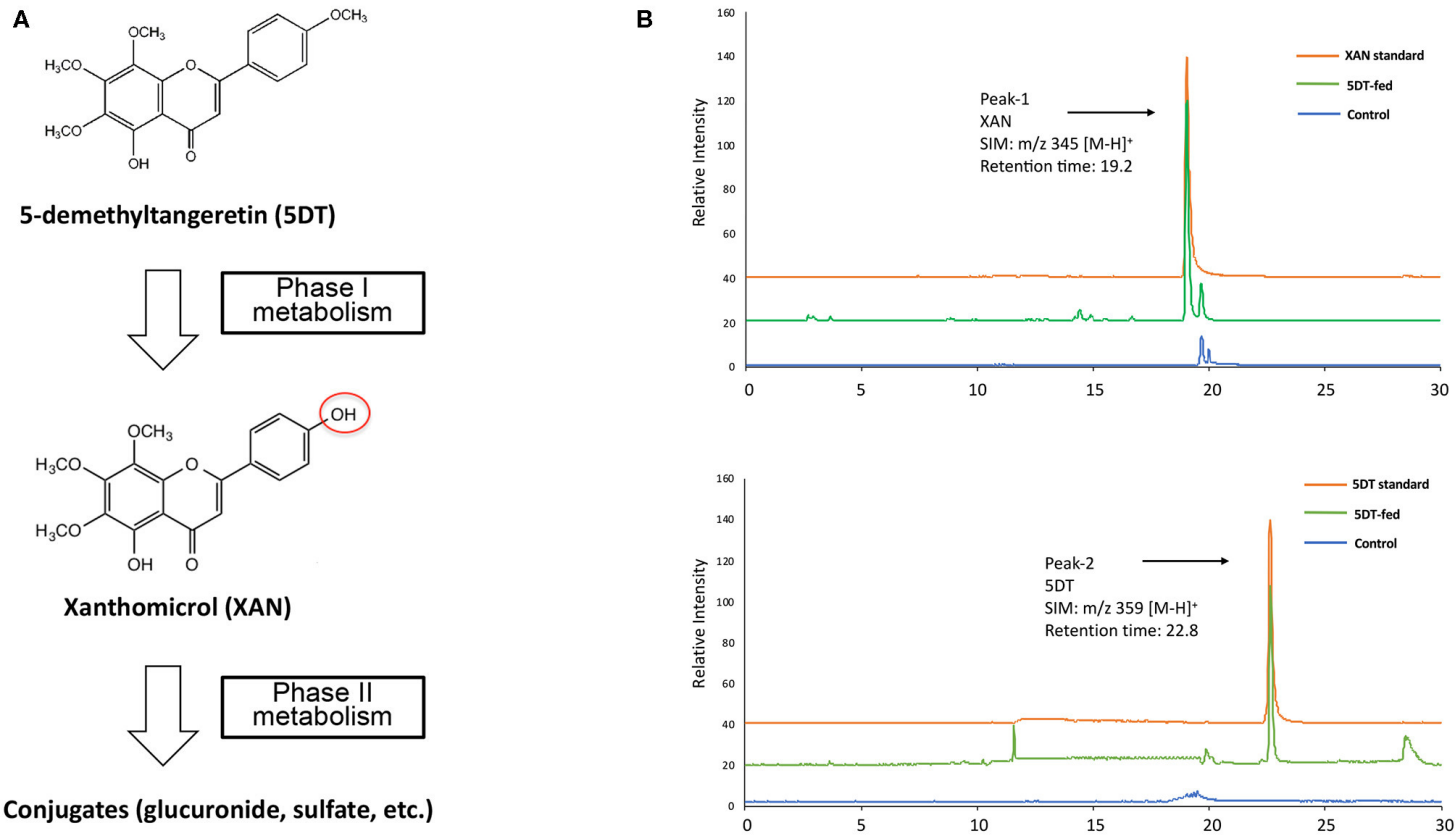
**(A)** Metabolic pathway of 5DT in mouse gastrointestinal tract. **(B)** Representative LC/ESI-MS chromatograms of synthesized standards for 5DT and XAN and the colonic mucosa from mice fed with or without 5DT.

As reported previously, XAN and 5DT were chemically synthesized, with purity >98% [Chemical structures are illustrated in Figure 1; (10, 18)]. All procedures and protocols were approved by the Institutional Animal Care and Use Committee of the University of Massachusetts (#2011-0066). Ten CD-1 mice (male, 5-week of age) were purchased from Charles River Laboratories (Wilmington, MA). All mice were kept in an air-conditioned room with 12-h light/night cycle, temperature of 23°C, humidity of 65–70%, and unrestricted access to AIN93G diet and water. After acclimation (1 week), animals were randomly assigned to one of the two groups (5 mice per group): (i) standard AIN93G diet and (ii) AIN93G diet supplemented with 0.1% 5DT (w/w). All mice were allowed to eat and drink *ad libitum*. After 10 weeks of intervention, all animals were sacrificed by CO_2_ asphyxiation. Colonic mucosa for LC-MS analysis was scraped and stored at −80°C.

### Identification of the Major Metabolites of 5DT in Mouse GI Tract

Samples for LC-MS analysis were prepared as we described previously (19, 20). Briefly, aliquots of the mucosa of mouse stomach, small intestine, cecum, and colon were homogenized with 50% methanol by Bead Ruptor Homogenizer (Omni International, Kennesaw GA). All sulfated and glucuronide metabolites were measured by enzymatic hydrolysis of the processed samples with β-glucuronidase and sulfatase as described (20, 21). Then the homogenates were extracted with equal volume of ethyl acetate for 3 times. The ethyl acetate extracts were combined and dried using a vacuum concentrator (Model: SVC 100H, Thermo Fisher Scientific Inc.), and then resuspend in 50% methanol for LC-MS (Model 2020, Shimadzu, Kyoto, Japan) analysis. Quantitation by LC-MS was performed by SIM, using ESI mode as described previously (22).

### Analysis of Cell Viability, Apoptosis, and Cell Cycle

HCT116 human colorectal cancer cells were purchased from American Type Cell Collection (ATCC, Manassas, VA). Assays for cell viability, apoptosis and cell cycle were performed as we previously described (7, 23, 24). Briefly, cells were seeded in a 96-well plate. After 24 h of incubation for cell attachment, HCT116 cells were treated with serial concentrations of XAN in serum complete media, and the cell viability was determined by MTT method (7, 23). HCT116 cells were seeded in a 6-well plate for apoptosis and cell cycle analysis. After 24 h, cells were treated with 15 and 21 μM of XAN, and after treatment, media containing any floating cells were harvested and combined with adherent cells that were detached by trypsin treatment. Cell pellets were then washed with ice-cold PBS and subject to apoptosis and cell cycle analysis using flow cytometry as we reported previously (7, 23).

### Immunoblot Analysis

Whole cell lysates of HCT116 cells were prepared using RIPA buffer with protease and phosphatase inhibitors (Boston BioProducts, Ashland, MA) according to the method we described previously (25–27). In brief, 50 μg proteins were separated on SDS-polyacrylamide gel electrophoresis (8 or 12%) and transferred onto a nitrocellulose membrane. After blocking, membrane was probed with different primary antibodies to p53, p21, cyclin D, and CDK4 (Cell Signaling Technology, Beverly, MA) at concentrations recommended by the manufacturer. Proteins were visualized and quantified using infrared imaging system (Odyssey CLx, LI-COR Biosciences, Lincoln, NE). β-Actin (Sigma-Aldrich, St. Louis, MO) was used as an equal loading control.

### Statistical Analysis

All data were presented as the mean ± SD or SE, unless otherwise specified. Student's *t*-test was used for the comparison between two groups. One-way analysis of variance (ANOVA) followed by Tukey's *post-hoc* test was used to test the mean difference between three groups. Statistical significance was accepted at a *P* < 0.05.

## Results and Discussion

### Metabolic Fate of 5DT in Mouse GI Tract

It is known that many flavonoids undergo extensive biotransformation and subsequently produce various metabolites as a result of the first-pass metabolism in the liver and GI tract. Interestingly, these metabolites may possess unique and even more potent biological activities. The detailed metabolic fate of 5DT in the GI tract has not been studied. Herein, we sought to characterize the metabolic fate of dietary 5DT in the GI tract of CD-1 mice, after long-term oral administration. 5DT was mixed with standard AIN93G diet at 0.1% (w/w). Mice in the 5DT intervention group were fed with AIN93G diet supplemented with 0.1% 5DT.

Body weight was monitored once a week, and the final weight of liver and spleen was recorded. There was no significant difference between control and 5DT-fed mice, with respect to body weight gain and the weight of liver and spleen. The percentage weight of spleen (spleen weight/ body weight) of control mice and 5DT-fed mice is 0.42 ± 0.03 and 0.38 ± 0.02, respectively. The percentage weight of liver of control mice and 5DT-fed mice is 3.58 ± 0.12 and 3.47 ± 0.15 mg, respectively. Further, no obvious appearance or behavioral difference was observed either, indicating no significant toxic effects associated with long-term dietary intake of 5DT in mice. It should be noted that the dose of 5DT used in this study is reasonably achievable in humans by taking dietary supplement of 5DT. This mouse dose is equal to approximately a dose of 750 mg of 5DT daily for a 60 kg adult based on the equivalent surface area dosage conversion factors (28), which warrants the translational implications of the present study.

Previously, we have determined the plasma levels of PMFs, such as nobiletin and 5DN (29, 30). We found that these PMFs and their metabolites were present in plasma at low concentrations; however, their bioavailability in the colon was drastically higher, reaching μM levels. We have reported that gut microbiota may play a critical role in the biotransformation of PMFs, thereby significantly enhanced their bioavailability in the colon (31, 32). Further, some metabolites of nobiletin and 5DN exerted greater suppressive effect on colon cancer cells than their parent compounds did (6, 16). Taken together, in this study, we chose only to focus on the metabolic fate of 5DT in the GI tract, and then determined the inhibitory of its major colonic metabolite on human colon cancer cells.

After a 10-week intervention, the GI mucosa samples obtained from both groups of mice were analyzed by LC-MS for identification and quantification of the metabolites of 5DT. We found that 5DT (SIM: m/z 359 [M-H]+, retention time of 22.8 min, Figure 1B Peak-2) underwent extensive biotransformation in 5DT-fed mice, and yielded multiple metabolites via phase I metabolism, and their sulfate and glucuronide conjugates via phase II metabolism. Among these metabolites, one metabolite (SIM: m/z 345 [M-H]+, retention time of 19.2 min, Figure 1B Peak-1) showed the highest abundance across different GI tissues. Based on its molecular weight, this metabolite was hypothesized to be xanthomicrol (XAN). Using chemically synthesized and authenticated XAN standard, we confirmed that the major metabolite of 5DT was XAN. This finding was consistent with previous reports showed that one major site of phase I metabolism of PMFs including nobiletin, tangeretin and 5DN, is the 4′-positions on B ring (6, 10, 13, 33, 34).

**Figure 2:**
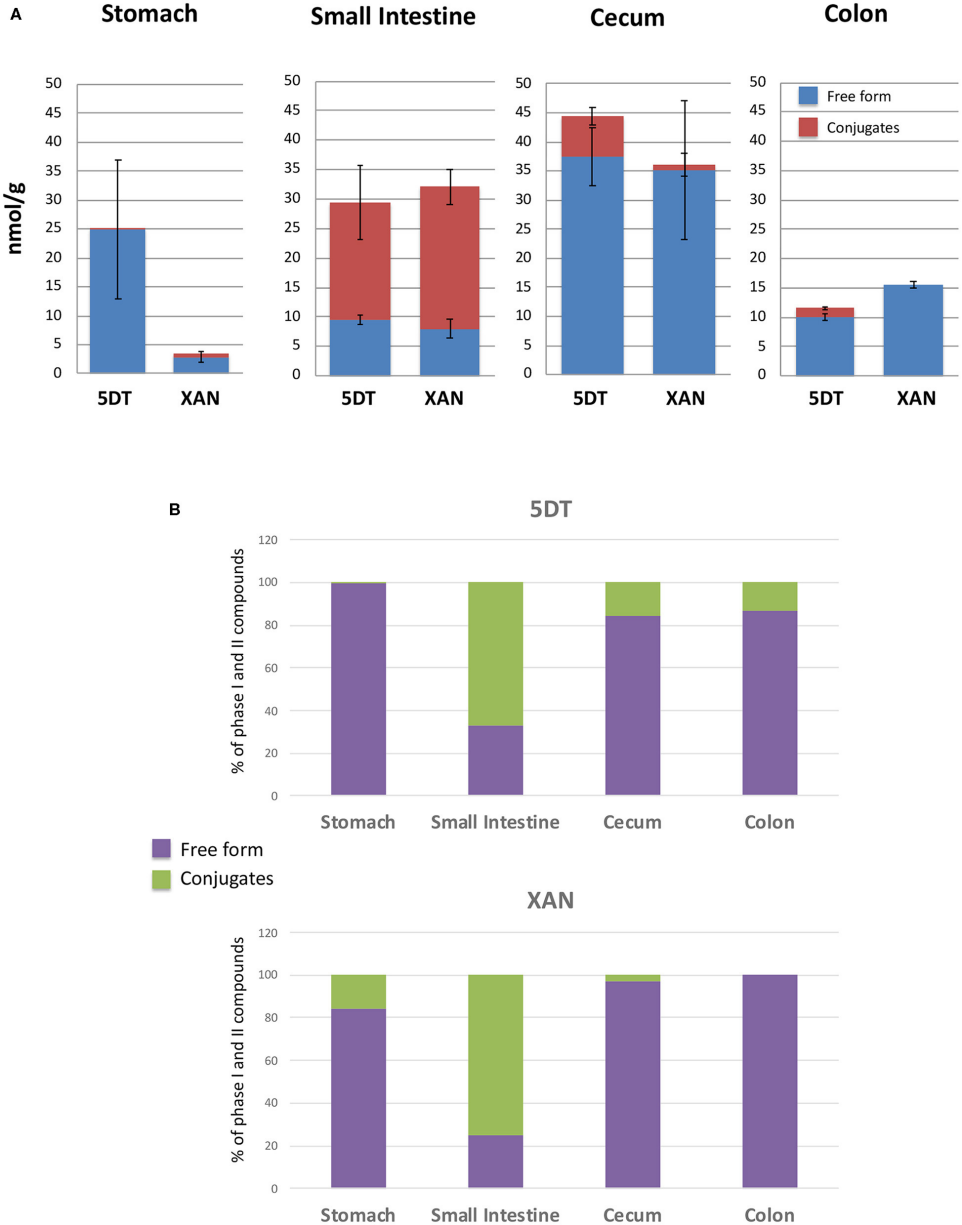
The absolute levels **(A)** and the percentage **(B)** of 5DT and XAN, and their conjugates in the stomach, small intestine, cecum, and colon of 5DT-fed mice. Data are shown as mean ± SD.

Phase II conjugation was mainly occurred in the liver and enterocytes that produce glucuronides, sulfates and methyl conjugates, and small amount of free aglycones (35, 36). In this study, we found that the phase II metabolites of 5DT were mainly presented in the small intestine, whereas a modest amount of phase II metabolites was also found in the cecum (Figure 2B). However, very limited amount of phase II conjugates was present in the colon. The drastic decrease in the percentage of phase II metabolites in the cecum and colon in comparison to the small intestine suggested that gut microbiota in the cecum and colon might help deconjugate these phase II metabolites of 5DT and transform them to the corresponding aglycons, e.g., from XAN glucuronide to XAN. Gut microbiota have a critical and complex role in transforming dietary phytochemicals. In fact, the health benefits of some phytochemicals including PMFs have been found to at least partially depend on their biotransformation in the gut (37, 38). Since phytochemicals and gut microbiome share a bidirectional relationship, 5DT and its colonic metabolites may impact the composition and function of gut microbiome as well. In future studies, the interplay between the two entities warrants further investigation.

### Quantification of 5DT and XAN in Mouse GI Tract

Since XAN is the primary metabolite of 5DT in GI tract of 5DT-fed mice, we further quantified the levels of 5DT, XAN, and their phase II conjugates in the colonic mucosa. Using the synthesized standards, we determined the levels of XAN and 5DT in mouse GI tract (Figure 2A). The concentrations of XAN and 5DT in the stomach were 2.88 ± 0.99 and 24.87 ± 12.04 nmol/g of tissue, respectively. The concentrations of XAN and 5DT in the small intestine were 8.04 ± 1.59 and 9.62 ± 0.80 nmol/g of tissue, respectively. The concentrations of XAN and 5DT in the cecum were 35.08 ± 11.95 and 37.47 ± 4.92 nmol/g of tissue, respectively.

The concentrations of XAN and 5DT in the colon were 15.59 ± 1.08 and 10.03 ± 0.65 nmol/g of tissue, respectively. We should note that the concentration of XAN was significantly lower than that of 5DT in the stomach and greater than that of 5DT in the colon. Interestingly, the concentrations of both 5DT and XAN achieved in the colon after 10-week dietary consumption were higher compared to those achieved after 4-week intervention of 0.1% (w/w) 5DT as we reported previously (17), suggesting that continued oral intake may further improve the bioavailability of 5DT. One of the underlying mechanisms could be improved composition of gut microbiota—many studies have shown that gut bacteria play an essential role in the biotransformation of dietary bioactive components, including PMFs (30, 31, 39). On the other hand, dietary compounds influence the composition of gut microbiota (40). Thus, we hypothesized that long-term dietary consumption of 5DT altered the composition of gut microbiota in mice, which in turn impacted the biotransformation of 5DT. Further research is warrant to understand the bidirectional relationship of gut microbiome and 5DT.

Using the 5DT and XAN standards, we further estimated the concentrations of their phase II metabolites in the GI tract. Briefly, only trace amount of XAN conjugates and 5DT conjugates were detected in the stomach and colon, respectively. The majority of phase II conjugates of 5DT and/or XAN were presented in the small intestine and cecum. In the small intestine, the concentrations of 5DT and XAN conjugates were ~19.73 ± 7.13 and 24.03 ± 4.64 nmol/g of tissue, respectively. And in the cecum, the concentration of 5DT conjugates was ~6.95 ± 3.35 nmol/g of tissue. It is very evident that the major forms of 5DT and XAN in the small intestine were the conjugated forms, which is in good agreement with previous studies (41).

### Inhibitory Effects of XAN, the Major Colonic Metabolite of 5DT, on HCT116 Human Colon Cancer Cells

**Figure 3:**
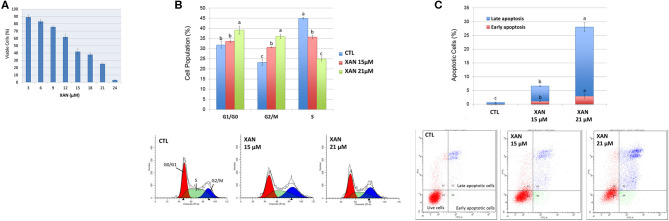
**(A)** Growth inhibitory effects of XAN on HCT116 human colon cancer cells. Effects of XAN (15 or 21 μM) on cell cycle progression **(B)** and apoptosis **(C)** of HCT116 human colon cancer cells. Data are shown as mean ± SD. Different notations in the bar charts indicate statistical significance (*p* < 0.05, *n* = 3) by ANOVA.

Colon cancer is one of the leading causes of cancer-related death in both sexes in the United States (42). The chemopreventive effects of 5DT have been reported in multiple models (7, 9, 43). However, the inhibitory effects of its major colonic metabolite on colon cancer cells remain unknown. To better understand the biological activities of 5DT, we determined the effects of XAN on the growth of human colon cancer HCT116 cell line, which is a widely-used *in vitro* model of colon cancer, and investigated the underlying molecular mechanisms. As shown in Figure 3A, overall, XAN exhibited a potent growth inhibitory effect on HCT116 cells. For example, XAN at 15 and 24 μM resulted in 58 and 97% inhibition, respectively, on HCT116 cells. Compared to our previous study on 5DT (7), although the growth inhibitory effect of XAN in HCT116 cells was not as strong as that of its parent compound 5DT, given the significantly greater level of XAN achieved in the colon after long-term consumption, it is still of great importance to investigate the bioactivities of XAN.

If we assumed that 1 gram of colonic tissue gave 1 mL of volume, the colonic level of XAN was 15.59 μM. It should be noted that this colonic concentration of XAN was achieved in mice by *ad libitum* feeding of standard diet containing 0.1% 5DT (w/w) throughout a 10-week feeding period. Thus, it reflects a stabilized and sustained level of XAN in the colon after long-term exposure to 5DT. If the mouse was given a one-time oral administration (e.g., via taking 5DT supplements in capsules), a much higher colonic level of XAN is likely to be achieved. To assess the suppressive effects of XAN on colon cancer in a physiologically relevant manner, 15 and 21 μM of XAN were chosen for the further experiments.

### Induction of Apoptosis and Cell Cycle Arrest by XAN

Next, the mechanisms of action of XAN in suppressing the growth of colon cancer cells were investigated. Uncontrolled cell cycle progression and reduced apoptosis are some common hallmarks of cancer (44), to determine if the induction of apoptosis and cell cycle arrest contributed to the growth inhibition of XAN, the effects of XAN on apoptosis and cell cycle of HCT116 cells were determined using flow cytometry. As shown in Figure 3B, 15 and 21 μM of XAN significantly increased cell accumulation in G2/M phase, while 21 μM of XAN also led to cell cycle arrest at G1/G0 phase. Moreover, XAN at 15 and 21 μM greatly induced both early and late apoptosis of HCT116 cells (Figure 3C). For example, XAN at 15 and 21 μM increased late apoptotic cell population by 9.2- and 41.8-fold in comparison to the untreated control cells, respectively. Taken together, the flow cytometric analysis indicated that activation of cell cycle arrest and cellular apoptosis was involved in the chemopreventive activities of XAN.

### Modulation of Key Signaling Pathways Associated Apoptosis and Cell Cycle by XAN Treatments

**Figure 4:**
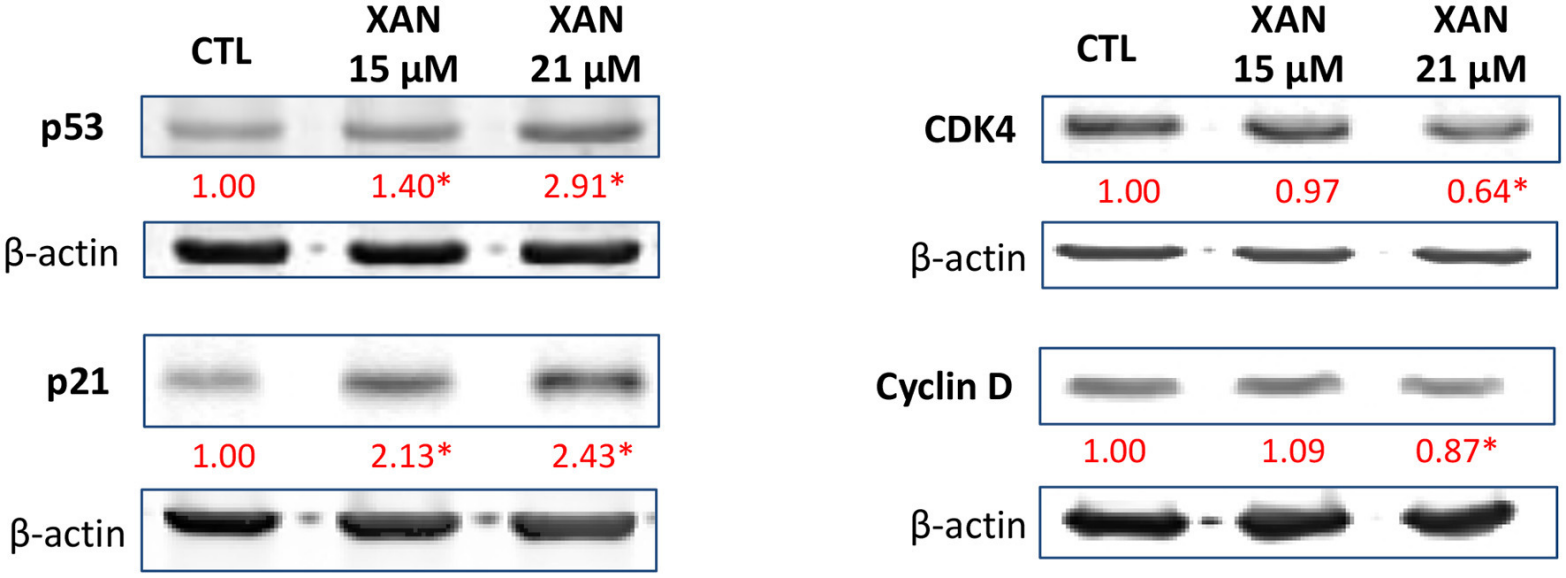
Effects of XAN (15 or 21 μM) on the protein levels of p53, p21, cyclin D, and CDK4 in HCT116 human colon cancer cells. β-Actin served as an equal loading control. The standard deviation (all within ±15% of the means) was not shown. *Indicates statistically significant difference compared to un-treated control group (*p* < 0.05, *n* = 3).

To further understand the role of XAN on apoptosis and cell cycle arrest, several key signaling proteins in these pathways were examined by western blot analysis. We found that XAN at 15 and 21 μM significantly upregulated the protein levels of p53 by 1.40- and 2.91-fold, and p21 by 2.13- and 2.43-fold, respectively (Figure 4). In colon cancer patients, p53 signaling is frequently dysregulated along with other pro-cancerous signaling such as *Wnt/*β-catenin and Ras (45). The p53 tumor suppressor has been extensively studied because of its critical role in antiproliferative processes. The expression of p53 can be triggered by DNA damage, hypoxia, and aberrant oncogene expression. A disrupted p53 therefore functions to promote genomic instability, defects in cell cycle checkpoints and survival of cancer cells (46). In HCT116 human colon cancer cells, p21 is expressed by a p53-dependent mechanism. P21 is a suppressor of cell cycle progression because it inhibits cyclin-dependent kinase (CDK)–cyclin complexes (47). Many CDKs and cyclins are overexpressed in human cancers, and some of them have been found to be required for tumor initiation and progression, such as cyclin D and CDK4 (48). In particular, different CDK-cyclin complexes function to promote cell cycle progression through different phases, for example, cyclin E/CDK2 and cyclin D/CDK4 regulate the cell-cycle during G1/S transition (48, 49). In this context, our studies demonstrated that subsequent to p53-dependent expression of p21 in the HCT116 cell treated with XAN at 21 μM, inhibition of expression of cyclin D and CDK4 occurred (Figure 4). Taken together, the western blot data was consistent with the observations from flow cytometry, suggesting that XAN attenuated the growth of HCT116 cells by inducing and arresting cell cycle progression.

Overall, the present study provided new information on the inhibitory effects of 5DT's primary metabolite XAN on colon cancer cells. In future, the relative potency of XAN in inhibiting colon cancer cells should be compared to that of a known anti-cancer drug to better understand the chemopreventive efficacy of XAN. *In vivo* study is needed to assess the bio-efficacy of XAN. Moreover, the role of gut microbiome in the biotransformation of 5DT remains unclear, which warrants future studies to determine the role of gut microbiota in metabolic fate of 5DT and XAN.

## Conclusion

In conclusion, for the first time, we investigated the metabolic fate of 5DT in mouse GI tract after long-term oral intake. We found that 5DT's 4′-position on the B-ring was a major site for demethylation reaction, resulting in the primary metabolite XAN. Moreover, we quantified the levels of XAN and 5DT in the mucosa of the stomach, small intestine, cecum, and colon. In addition, we found that XAN exerted significant growth inhibitory effects on HCT116 human colon cancer cells at physiologically achievable concentrations via inducing apoptosis and cell cycle arrest. This study provided useful information on the biological activity and mechanism of action of 5DT, XAN, and other related PMFs.

## Data Availability Statement

The original contributions presented in the study are included in the article/supplementary material, further inquiries can be directed to the corresponding author/s.

## Ethics Statement

The protocol for the animal experiment was approved by the Institutional Animal Care and Use Committee of the University of Massachusetts (#2011-0066).

## Author Contributions

XW and HX conceived and designed all experiments, analyzed the data, and wrote the manuscript. XW, ZL, YS, FL, ZG, and JZ performed the experiments. All authors read and approved the final manuscript. All authors contributed to the article and approved the submitted version.

## Conflict of Interest

The authors declare that the research was conducted in the absence of any commercial or financial relationships that could be construed as a potential conflict of interest.

## Abbreviations

PMF: polymethoxyflavone
TAN: tangeretin
5DT: 5-demethyltangeretin
XAN: xanthomicrol.
